# Microbothriid (Monogenean) Infection and Treatment in Captive Blacktip Reef Shark (*Carcharhinus melanopterus*)

**DOI:** 10.3390/microorganisms12122558

**Published:** 2024-12-12

**Authors:** Hee Jun Ko, Sung Bin Lee, Kee Hoon Kim, So Young Jeon, Ji Yun Jung, Yun Mi Choi, Se Chang Park

**Affiliations:** 1 Laboratory of Aquatic Biomedicine, Research Institute for Veterinary Science, College of Veterinary Medicine, Seoul National University, Seoul 08826, Republic of Korea; hjk9538@gmail.com (H.J.K.); lsbin1129@snu.ac.kr (S.B.L.); 2 Lotteworld Aquarium, Seoul 05551, Republic of Korea; keehoonk@lotte.net (K.H.K.); sy.jeon@lotte.net (S.Y.J.); jyjung@lotte.net (J.Y.J.); yunmi.choi@lotte.net (Y.M.C.)

**Keywords:** microbothriid, monogenean, blacktip reef shark, aquarium, microstructure, SEM, opisthaptor, treatment, trichlorfon

## Abstract

Unlike other microbothriid monogenean infections in elasmobranchs, limited information is available on the biology and treatment of *Dermophthirius melanopteri*. As parasitic infection with *D. melanopteri* was found in 21 juvenile blacktip reef sharks (*Carcharhinus melanopterus*) at the Lotteworld aquarium in Seoul, South Korea, we aimed to investigate the anatomical features and treatment protocols for *D. melanopteri* in this study. The parasites were sampled and fixed in 10% neutral-buffered formalin, and examined using light and scanning electron microscopy. The treatments included short-term praziquantel baths, freshwater immersion, and long-term trichlorfon baths. Manual removal of parasites was also attempted. Examination and manual removal revealed that, similar to other microbothriids, *D. melanopteri* attaches to the placoid scale using opisthaptoral secretion in the posterior region. The effectiveness of the treatments varied, with trichlorfon proving the most effective and safe option for complete parasite eradication. Praziquantel facilitated parasite removal by weakening their attachment, suggesting the potential for higher doses and prolonged exposure times for enhanced antiparasitic effects. To our knowledge, this is the first study providing the microscopic details of *D. melanopteri* infection and its treatment outcomes in captive blacktip reef sharks, thereby providing valuable insights for future research and management.

## 1. Introduction

Infections caused by microbothriid monogenean parasites are common among elasmobranchs in aquaria [1,2,3,4,5]. Monogeneans are parasitic flatworms predominantly inhabiting fish, distinguished by their remarkably high host specificity [6]. These parasites exhibit remarkable morphological diversity, with significant variations in size, shape, and the intricate mechanisms they use for anchoring to their hosts [7]. Within this group, the *Dermophthirius* genus represents a collection of small parasitic monogeneans that employ a specialized attachment organ called an opisthaptor to firmly secure themselves to the host’s skin and gills [2,3,4]. By parasitizing and consuming the host’s epithelial cells, *Dermophthirius* inflicts substantial damage, often manifesting as skin lesions or ulcerations that can critically compromise fish health [8,9]. The potential of these parasites to dramatically impact fish populations renders them a vital subject of extensive research in aquarium management and aquaculture settings. In the early stages of infection, hosts show behavioral changes such as erratic swimming and rubbing [8]. Without appropriate treatment, they may lose their placoid scales and develop ulcerated skin lesions, leading to secondary bacterial infections [8,9].

The blacktip reef shark (*Carcharhinus melanopterus*) is one of the sharks commonly found in public aquarium exhibits worldwide because of their stereotypical ‘shark-like’ appearance and relatively small size, reaching approximately 120 cm in size when fully grown [10]. Parasitic infections associated with blacktip reef sharks have been previously studied. An infection with *Huffmanela selachii*, a skin nematode, in wild blacktip reef sharks was reported in Saudi Arabia [11], while Burnet Park Zoo in the United States reported a case of myxocystis infection in the skeletal muscles of captive blacktip reef sharks [12]. However, except for *Dermophthirius melanopteri*, microbothriid infection has not been reported in blacktip reef sharks [13].

Monogenetic trematode infestation was observed in blacktip reef sharks at Lotteworld aquarium, South Korea. Based on the morphological features, small size of the opisthaptor, absence of hooks, and host specificity, the parasite was identified as *D. melanopteri*. To date, there have been no reports of treatment methods for *D. melanopteri*. *Neodermophthirius* sp., which belongs to the same family as *D. melanopteri*, has been successfully treated using copper sulfate [5]. Further, *Dermophthirius* sp. infection in captive lemon sharks has been successfully treated with formalin and trichlorfon; however, pathogenicity and susceptibility to treatment may differ among species [5,8]. In addition, the ability of host species to tolerate chemical agents may differ, leading to adverse reactions [14,15].

This study describes the characteristics of *D. melanopteri* and its infection. In this study, we describe the appearance and anatomical features of this parasite and demonstrate its infection characteristics in 21 juvenile blacktip reef sharks.

## 2. Materials and Methods

### 2.1. Study Object

In total, 21 juvenile blacktip reef sharks were examined (Table 1). Of these, 11 were brought into the aquarium in 2020, and 10 were brought in 2022. More specific information is provided in Table 1. Although their introduction times were different, they had a history of being maintained in the same tank. The tank that all individuals visited in common was a quarantine tank that shared water with a large exhibition tank, and various species such as zebra sharks (*Stegostoma tigrinum*), whitetip reef sharks (*Triaenodon obesus*), giant guitarfish (*Rhynchobatus djiddensis*), bowmouth guitarfish (*Rhina ancylostoma*), and other teleosts were placed together in this large tank. The water quality parameters in this tank were maintained as follows: temperature, 23.5–24.5 °C; pH, 7.4–7.8; and salinity, 28–32 ppt.

### 2.2. Parasite Sampling, Morphological Analysis, and Species Identification

Parasite sampling was performed during bath treatment by manual removal using a razor without administering anesthesia. Among the parasites removed from the infected sharks, those with the most intact shape were placed in 10% neutral-buffered formalin for further analysis. The samples were then placed on glass slides and observed under a clinical microscope BX41 (Olympus, Center Valley, PA, USA) at 10× magnification for morphological examination. ImageJ software version 1.53 was used for measuring the parasites [16].

Morphological features such as the small size of the opisthaptor, absence of hooks, and host specificity precisely matched those of *D. melanopteri* among the parasites previously reported in blacktip reef sharks [13].

### 2.3. Scanning Electron Microscopy (SEM)

Specimens were prepared for scanning electron microscopy (SEM) according to the following protocol [17]. The fixed sample in 10% neutral-buffered formalin was first washed with 0.05 M sodium cacodylate buffer and then immersed in a solution of 1% osmium tetroxide diluted in 0.1 M sodium cacodylate buffer for 2 h. The samples were then rinsed thoroughly with distilled water. Dehydration was gradually performed using ethanol concentrations of 30%, 50%, 70%, 80%, 90%, and 100% for 20 min in each step, and a critical point dryer (CPD), model EM CPD300 (LEICA Biosystems, Wetzlar, Germany) for 2 h. A sputter coater, specifically an EM ACE200 (LEICA Biosystems, Wetzlar, Germany), was used to deposit a 20 nm platinum layer onto the dried sample. Morphological examinations were conducted using a field-emission scanning electron microscope (FESEM), model SIGMA (Carl Zeiss, Oberkochen, Germany). When observed from a distance, the working distance was 11.9 mm and the operating voltage was 5.0 kV. When observed with magnification, the working distance was 5.0 mm and the operating voltage was 10.0 kV.

### 2.4. Treatments

Infected blacktip reef sharks were subjected to at least one of the following treatments: a short-term praziquantel bath, freshwater immersion, or a long-term trichlorfon bath. For the short-term praziquantel bath, an isolated bathing space was prepared by transferring water to a 1000 L jumbo box with aeration. Each shark was moved to a jumbo box using fishing nets of suitable sizes. Considering the size and characteristics of the blacktip reef sharks, up to four sharks were isolated in one jumbo box for treatment to minimize their stress. They were left in the box for several minutes to adapt to the space. After stabilization, praziquantel was administered at 15–20 ppm per treatment and the sharks were immersed for up to 1 h. When any abnormal symptoms, such as erratic swimming, were observed, the treatment was immediately stopped and the sharks were returned to the original captive tank. After each treatment, the efficacy of praziquantel was evaluated based on the number of parasites.

For freshwater immersion, a 1000 L jumbo box was prepared in the same manner as the praziquantel treatment, in which freshwater was filled. The infected sharks were transferred using a fishing net, with each shark exposed to freshwater for no more than 5 min. Whenever the condition of the sharks deteriorated, they immediately returned to the original seawater. The treatment efficacy was monitored after each treatment.

For long-term treatment, the sharks were moved to a treatment tank (25 tons, 5 × 2.5 m). The water temperature was set at 26.5 °C and the organophosphate trichlorfon was used for long-term treatment. A single treatment session comprised two to three trichlorfon administrations at 3-day intervals. The initial concentration of administration was 0.15 ppm, but this was raised to 0.2 ppm after determining the tolerance of the sharks. The effect of the treatment was monitored, and if necessary, the session was repeated at intervals of approximately 3 weeks. Considering the characteristics of trichlorfon [8], it was administered in the afternoon with the light off, and the skimmer was shut down for approximately 12 h immediately after administration.

Further, manual removal of parasites was attempted during some treatments. The infected sharks were lifted using a fishing net to expose the infected area to air. Next, the adhesive organ of the parasite was scraped off using a sterilized razor in the same direction as the scales of the host. Because blacktip reef sharks are obligate ram ventilators, one rest period was given after removing one parasite. This procedure was repeated until almost all visible parasites were eliminated. After parasite removal, recurrence and symptom improvement were monitored.

## 3. Results

### 3.1. Case History and Symptoms

**Figure 1:**
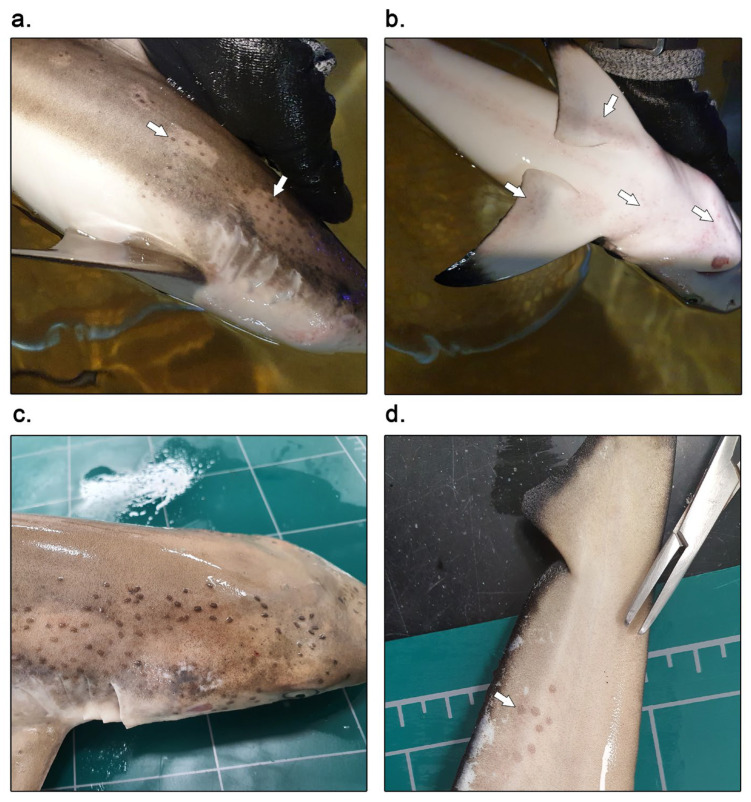
The monogenean *Dermophthirius melanopteri* infecting the blacktip reef shark (*Carcharhinus melanopterus*). (**a**,**b**,**d**) Numerous black or grey spots on the skin of the blacktip reef shark, which were determined as *D. melanopteri* (white arrows). The parasites were observed particularly on the dorsolateral (**a**) and ventral (**b**) part of the head and body (white arrows). Few parasites were also attached on the tail (**d**) (white arrow). (**a**,**c**) White discolored focal patches and ulcerative lesions were seen around the eyes and gill slits.

The first onset of symptoms was observed in BRS1, BRS2, BRS3, BRS4, BRS5, BRS6, and BRS7. One month and three weeks after introduction to the aquarium, black-to-gray oval-shaped spots were spread all over the sharks’ skin (Figure 1). They were observed particularly on the surface of the head and gill slits (Figure 1c), as well as in other areas such as the dorsal fin, dorsoventral part of the body (Figure 1a,b), and tail (Figure 1d). Focal discoloration and erosion (Figure 1a,c) with erythema were observed in areas where an excessive number of trematodes were attached. Other clinical signs such as pruritus and loss of appetite were observed. Pruritus was indicated by behavioral changes, including rubbing against the tank floor.

The same symptoms were observed in the sharks brought in afterward. The time required for symptom onset was also similar. BRS8, BRS9, BRS10, and BRS11 were found to have the same symptoms as those of the previous sharks 52 days after being brought in. In the 10 blacktip reef sharks brought in 2022 (BRS12, BRS13, BRS14, BRS15, BRS16, BRS17, BRS18, BRS19, BRS20, BRS21), the same symptoms were observed even faster, i.e., less than a month after they were brought in.

### 3.2. Parasite Morphology

**Figure 2:**
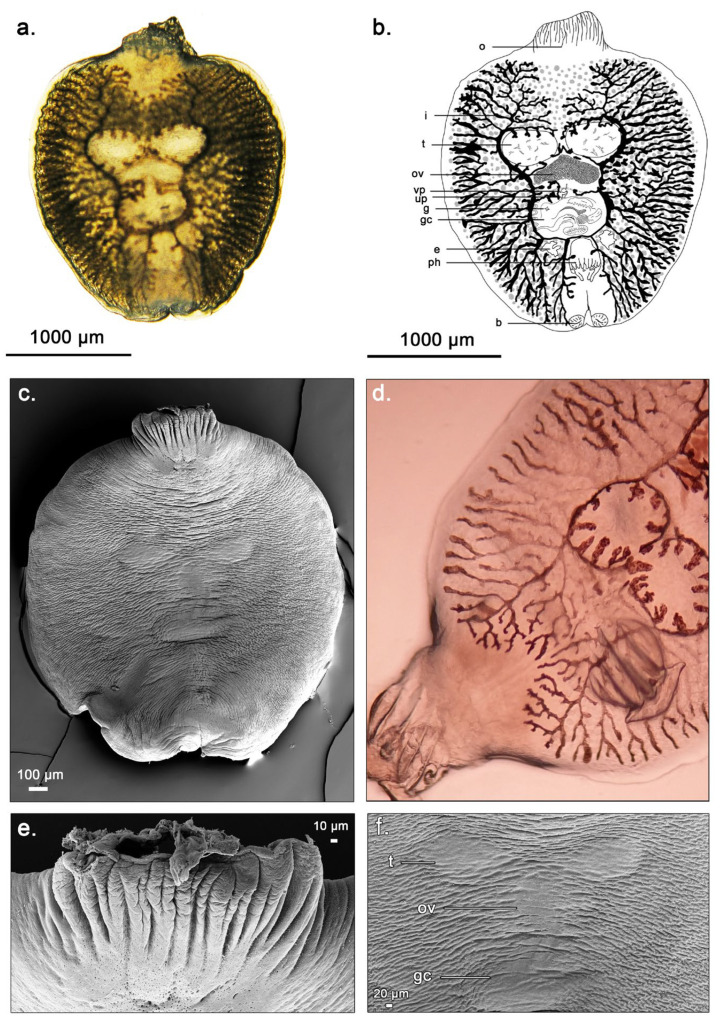
Morphologic analysis of the parasite *Dermophthirius melanopteri* infecting the captive blacktip reef shark (*Carcharhinus melanopterus*). (**a**) Structure of *D. melanopteri* captured using a light microscope. (**b**) Diagram of the ventral view of the internal organs of *D. melanopteri*, illustrated by S.B. Lee, referencing a previous study (Cheung et al., 1988) [13]. (**c**) Scanning electron microscopy (SEM) result of the whole parasite mount. (**d**) The enlarged light microscope image of the posterior part of the parasite. Note some placoid scales attached on the opisthaptor of the parasite. (**e**) The enlarged SEM photograph of the opisthaptor part of the parasite. (**f**) The enlarged SEM photograph of the reproductive organs of the parasite. Abbreviations: b, bothridia; e, excretory organ; g, genital pore; gc, genital complex; i, intestine; o, opisthaptor; ov, ovary; ph, pharynx; t, testes; up, uterine pore; and vp, vaginal pore.

The parasite had an oval-shaped, flat body, and was transparent in that, the internal organs were visible. The body measured 2.4 mm in length and 1.0 mm in width, consistent with the morphology observed in the previous examination (Figure 2a) [13]. The pharynx and genital complex occupied the anterior part of the body, whereas the posterior part was filled with the ovary, two testes, and opisthaptor (Figure 2b). The intestines were scattered around the lateral part of the body, and the two excretory organs were located laterally in the anterior part. In addition, a vaginal pore, uterine pore, genital pore, and two bothridia were observed on the body of the parasite. The opisthaptor, which had no hooks, measured 263.1 μm in length and 547.8 μm in width. Paired testes measured 267.5 μm and 271.1 μm in length, and 443.8 μm and 446.8 μm in width, respectively. The heart shaped ovary was anterior to the testes, and measured 195 μm in length and 542.2 μm in width. The genital complex measured 305 μm in length and 513 μm in width. Lastly, the pair of bothridia measured 106.7 μm and 107.2 μm in length, and 147.4 μm and 175.5 μm in width, respectively.

In the SEM results (Figure 2c), the surface of the parasite clearly revealed the presence of the opisthaptor (Figure 2e) and reproductive organs, including the ovaries, testes, and genital complex (Figure 2f).

### 3.3. Effect of Treatments

The effectiveness of treatments for infected blacktip reef sharks varied depending on the method used. Short-term praziquantel treatment was administered to BRS1, BRS2, BRS3, BRS4, BRS5, BRS6, and BRS7. After treatment with praziquantel, no parasites fell off of the sharks, and neither spots on the infected area nor loss of appetite were improved. No other adverse events were observed (Table 2).

**Figure 3:**
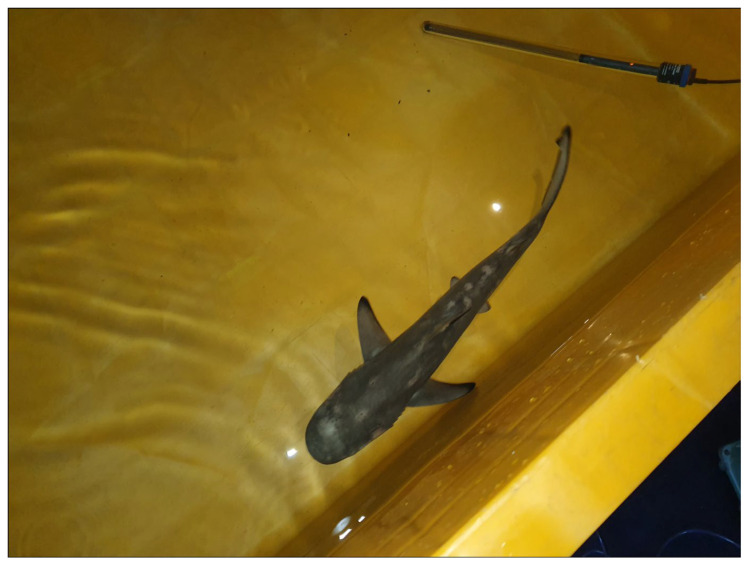
Freshwater immersion performed on infected blacktip reef sharks. The electric water heater was used to maintain the water temperature during treatment.

Upon freshwater immersion of infected blacktip reef sharks (Figure 3), no immediate effects were observed. However, a few minutes after treatment, the number of trematodes visible to the naked eye decreased significantly.

In some cases, manual removal was used concurrently with praziquantel treatment or freshwater immersion. In the case of freshwater immersion, even though a razor was used during removal, the trematode haptor did not completely separate from the host scale, and separation was only achieved using a significant force that was sufficient to damage the haptor or scales. However, manual removal during praziquantel treatment allowed trematodes to fall off more easily without damaging either the parasite or the host. After removal, a temporary decrease in the number of parasites and a reduction in the size of skin discoloration were observed. Complete removal of all parasites during a single treatment session was not possible because of the stress induced in the sharks during handling, which limited the duration and intensity of the procedure. Shortly after, a decrease in host immunity resulted in a visible increase in the number of pathogens. Further, despite the rest periods between physical removal sessions, some sharks such as BRS8 and BRS9 were unable to withstand the stress of capture and showed stress responses during immersion. Even after returning to the original tank, they did not swim, but instead sank to the bottom, breathing temporarily, and eventually dying after a few minutes.

Trichlorfon treatment was first applied to BRS12, BRS13, BRS14, and BRS15 on a trial basis because trichlorfon is known to be fatal to elasmobranchs when misused. They showed no abnormalities after treatment; instead, the number of pathogens dramatically decreased when observed with the naked eye. Recurrence, such as an increase in parasite number, occurred after the second trial in February 2022 and the third trial in March 2022; however, the number of parasites visibly disappeared following each subsequent treatment session, with complete clearance observed within a few days of treatment. Through multiple treatment sessions, the period between recurrences became longer, and after the last treatment session conducted in June 2022, the parasites were completely eradicated. No deaths occurred during any trichlorfon treatment process.

## 4. Discussion

To date, only one brief description regarding the morphology of the posterior adhesive organ of *D. melanopteri* has been reported [13]. Cheung et al. described the opisthaptor of *D. melanopteri* as a muscular sucker resembling a cup. If this is the only method used by *D. melanopteri*, the parasite should be dislodged by inserting a sharp material under the edge of the opisthaptor [18]. However, in our study, live *D. melanopteri* could not be dislodged in this manner. Detachment of the parasite was forcible, and even when it detached from the host, the scales of the host remained attached to the adhesive organ (Figure 2d). A similar method of attachment has been observed in *Leptocotyle minor* and *D. carcharhini*, both of which belong to the same family as *D. melanopteri* [1,2]. Kearn found that *L. minor* attaches itself to the surface of the host denticle not by suction, but by cement secreted from the cement gland located in the posterior region of the body of the parasite [1]. Rand et al. investigated the components of opisthaptoral secretion using histochemical analyses and found that the material showed a strong positive reaction in Millon’s tyrosine test and the Sudan IV lipid test [2]. Thus, the presence of a secretory gland connected to the opisthaptor is strongly suspected in *D. melanopteri,* even with the lack of staining with Ehrlich’s hematoxylin [1]. In the present study, an organ with an appearance similar to the cement gland mentioned in a previous study [1] was also found in the posterior region of the body near the adhesive surface of the opisthaptor.

The observed clinical signs, such as skin discoloration, are likely due to the attachment and feeding mechanism of *D. melanopteri* because parasites were more frequently found on lesions than on normal skin. *D. melanopteri* belongs to Monopisthocotylea, which are characterized by feeding on epithelial cells [19]. This was confirmed in a study on skin lesions caused by *D. penneri*, a parasite with the same genus as *D. melanopteri* [9]. According to Bullard et al., *D. penneri* can only access the skin epithelial cells and cannot harm the underlying tissues [9]. Because placoid scales consist of a denticle, it is likely that the epithelium is the only source of nutrition for the parasites. In this feeding process, digestive enzymes, such as proteases and phosphatases, may affect the host epithelium [20] and cause skin damage. Further, *D. melanopteri* may move from one attached denticle to another using the bothridia located on the anterior part of the parasite [1,6]. The ability to move on the host body allows the parasite to further expand the skin lesions.

As an organophosphate, trichlorfon may provoke sensitive reactions in elasmobranchs, particularly in eagle rays (*Aetobatus* spp.) and guitarfish (*Rhinobatos* spp. and *Rhina* spp.) [21]. Side effects, including alteration of skin color, inappetence, and mortality, have been reported in a study conducted with cownose rays (*Rhinoptera bonasus*) [22]. Although trichlorfon has been reported to be successfully used against *Dermophthirius* sp. in captive lemon sharks [8], there was no proof that the same result would be obtained with blacktip reef sharks, owing to the lack of relevant studies in these sharks. In the present case, trichlorfon showed no apparent side effects or mortality and was the most effective and safe treatment among those tested. Furthermore, unlike the study by Cheung, which used 0.5 ppm of trichlorfon at 1-week intervals for 3 weeks [8], a maximum 0.2 ppm with continuous exposure and up to two more applications at 3-day intervals was effective enough to eliminate *Dermophthirius* sp. in blacktip reef sharks. Although efficacy may differ between monogenean species and hosts, our results suggest a lower but possibly more effective dose of treatment, considering the harmful threat that trichlorfon poses to humans [23].

Praziquantel is generally known to be effective in exterminating monogeneans [15,24,25,26,27,28,29]. However, in our study, praziquantel did not exhibit immediate antiparasitic effects, but appeared to facilitate the physical removal of parasites by weakening their attachment to the host. According to Norbury et al., higher praziquantel doses are required to remove the trematodes attached to the skin than those required for blood-feeding trematodes [30]. Further investigation of higher doses and prolonged exposure times of praziquantel is needed to determine whether these adjustments could yield more significant antiparasitic effects on *D. melanopteri*. In addition, the possibility that praziquantel affects the adhesive glands of trematodes should be considered. The mechanism by which praziquantel acts against trematodes remains unclear. However, praziquantel has been proposed to bind to voltage-gated calcium channels and increase calcium ion flux into the fluke [30,31]. This mechanism may be related to the loosened attachment of the parasite; therefore, additional research is needed on the attachment-related components of monogenean trematodes.

Although manual removal of skin trematodes is one of the most reliable methods for eliminating adult parasites, it is risky to perform without anesthesia. Because sharks maintain strong wildness, they would experience severe stress regarding survival when physically restrained without anesthesia. According to Wosnick et al., when elasmobranchs encounter excessive stress, muscle stiffness may occur because of a physiological bioadaptive response [32]. Excessive handling and physical restraint can lead to stress-induced muscle stiffness and lactic acidosis, potentially resulting in mortality. In the present case, BRS8 and BRS9 showed sudden muscle stiffness and loss of mobility after physical restraint. After they sank to the bottom, artificial swimming was induced by lifting them, as recommended by Wosnick et al. [32]. At first, they showed mild voluntary movement and the stiffness seemed to be relieved; however, they sank again and eventually died. Because blacktip reef sharks are obligate ram ventilators, loss of mobility indicates a lack of oxygen supply to the gills.

Notably, only juvenile sharks were infected by the same parasites identified in this case. In addition to the 21 sharks mentioned in this case, animals of the same species previously kept in the same tank as the subjects did not show any symptoms. Further, infection with the same parasites was observed in the subadult sharks brought in after this case, but the symptoms were not severe; therefore, no treatment was administered. By observing them without treatment until they became adults, we found that the parasite infection resolved naturally. This may be related to the growth level and immunity of the host organism; however, additional studies are required to confirm this. Finally, this study had the limitation of not conducting genomic analysis of this parasitic species, as we focused on individual treatment of the sharks and morphological analysis. Since genomic analysis of this species has not yet been sufficiently performed, developing primers for amplifying the partial sequence of *D. melanopteri* and conducting phylogenetic analysis remain priority tasks for the future. Further genomic research could provide additional insights into *D. melanopteri* and would be a valuable resource for the world community of parasitology.

## Figures and Tables

**Table 1:** Detailed information about captive blacktip reef sharks (*Carcharhinus melanopterus*) infected with the microbothriid *Dermophthirius melanopteri*.

Shark	Introduction Date	Age Group	Size (cm)
BRS1	March 2020	Juvenile	60
BRS2	March 2020	Juvenile	60
BRS3	March 2020	Juvenile	61
BRS4	March 2020	Juvenile	63
BRS5	March 2020	Juvenile	60
BRS6	March 2020	Juvenile	58
BRS7	March 2020	Juvenile	61
BRS8	July 2020	Juvenile	63
BRS9	July 2020	Juvenile	64
BRS10	July 2020	Juvenile	59
BRS11	July 2020	Juvenile	62
BRS12	January 2022	Juvenile	51
BRS13	January 2022	Juvenile	52
BRS14	January 2022	Juvenile	50
BRS15	January 2022	Juvenile	50
BRS16	February 2022	Juvenile	49
BRS17	February 2022	Juvenile	53
BRS18	February 2022	Juvenile	52
BRS19	February 2022	Juvenile	50
BRS20	February 2022	Juvenile	54
BRS21	February 2022	Juvenile	50

**Table 2:** Treatments administered to captive blacktip reef sharks (*Carcharhinus melanopterus*) infected with the microbothriid *Dermophthirius melanopteri*. Treatments are presented in chronological order.

Date	Treatment	Dose (ppm)	Frequency	Duration (hr)	Effectiveness	Shark
April 2020	Praziquantel	15	Single	1	No	BRS1–BRS7
May 2020	Praziquantel	20	Single	1	No	BRS1, 2
	Praziquantel + manual removal	20	Single	1	Partial	BRS3, 4
	Praziquantel + manual removal	20	Single	1	Partial	BRS1–BRS6
June 2020	Praziquantel + manual removal	15	Single	1	No	BRS1–BRS6
	Praziquantel + manual removal	20	Single	1/3	Partial	BRS1–BRS6
September 2020	Freshwater	-	Single	1/6	Partial	BRS11
October 2020	Freshwater	-	Single	1/6	Partial	BRS8–BRS11
November 2020	Freshwater + manual removal	-	Single	1/6	Partial	BRS8–BRS11
December 2020	Freshwater + manual removal	-	Single	1/6	Partial	BRS8–BRS11
January 2022	Trichlorfon	0.15–0.2	q3d, twice	24	Yes	BRS12–BRS15
February 2022	Trichlorfon	0.15–0.2	q3d, 3 times	24	Yes	BRS12–BRS21
March 2022	Trichlorfon	0.2	q3d, 3 times	24	Yes	BRS12–BRS21
June 2022	Trichlorfon	0.2	q3d, twice	24	Yes	BRS12–BRS21

## Data Availability

The original contributions presented in the study are included in the article, further inquiries can be directed to the corresponding author.
